# Medical Technology Transfer and Health System Capacity Building in Pakistan: Evidence from the China–Pakistan Economic Corridor

**DOI:** 10.12669/pjms.42.4.16011

**Published:** 2026-04

**Authors:** Zhang Tong, Qu Qiumei

**Affiliations:** 1 Zhang Tong Lecturer, College of Humanities, Heilongjiang Dongfang University, Harbin, China; 2 Qu Qiumei Visiting Lecturer, Department of History & Pakistan Studies, University of the Punjab, Lahore, Pakistan

**Keywords:** China-Pakistan Economic Corridor, Medical Technology Transfer, Health System Capacity Building, Hardware Output, Joint System Development

## Abstract

As part of the Belt and Road Initiative, the China-Pakistan Economic Corridor (CPEC) has gradually broadened the scope of its collaboration from the first energy and infrastructure projects to include human development and health. This study examines medical technology transfer and health system capacity building in Pakistan within the framework of CPEC. It details the current state of Pakistan’s healthcare system, including the absence of a comprehensive healthcare infrastructure and health insurance system, the lengthy and costly training cycles for medical professionals, Pakistan’s relatively insufficient investment in medical education, and the uneven distribution of healthcare resources between urban and rural areas. It proposes strengthening Sino-Pakistani medical cooperation through measures such as facilitating medical technology transfer between the two countries, prioritizing capacity building in Pakistan, and leveraging China’s indirect role in promoting rule of law. This approach aims to transition the China-Pakistan Economic Corridor from “hardware export” to “joint institutional development.

## INTRODUCTION

The Belt and Road Initiative is an international economic cooperation initiative proposed by President Xi Jinping in 2013, serving as the abbreviated term for the Silk Road Economic Belt and the 21st Century Maritime Silk Road.^1^ CPEC is a flagship project under the Belt and Road Initiative jointly developed by China and Pakistan. It was formally proposed during Premier Li Keqiang’s visit to Pakistan in May 2013. By the end of 2013, when President Xi Jinping put forward the Belt and Road Initiative, CPEC emerged as a valuable complement to the broader initiative, further elevating its strategic significance.

CPEC is a development belt and growth axis that offers mutual benefits, cooperation, complementary advantages, and shared prosperity. CPEC is based on large-scale cooperative projects for infrastructure construction, industrial development, and livelihood improvement, with the goal of socioeconomic development, prosperity, and security in the regions along it. Its main axis is the comprehensive transportation corridor and industrial cooperation between China and Pakistan, and its engine is people-to-people exchange and cultural communications, along with tangible economic and trade cooperation. 2013–2015 served as the initial phase of CPEC, during which the primary focus was on reaching agreements and designing the institutional framework.^2^ In April 2015, President Xi Jinping emphasized that CPEC serves as a crucial vehicle for achieving shared development between China and Pakistan. He proposed a “1+4” cooperation framework centered on corridor construction, with key focus areas including Gwadar Port, energy, infrastructure development, and industrial collaboration.

### The Five Corridor &”5Es”

In December 2017, CPEC Master Plan (2017–2030) aligned China’s Belt and Road Initiative with Pakistan’s Vision 2025, directing corridor development toward connectivity, energy, trade, and industrial parks. In 2020, Gwadar Port commenced operations as the flagship project of the corridor. In summary, the period from 2015 to 2020 served as the infrastructure development phase, emphasizing a cooperation model centered on corridor construction with a focus on infrastructure development.^3^ Since 2021, both sides have reached consensus on deepening the alignment between the “Five Corridors” (growth, livelihood, innovation, green, and openness) and the “5Es” development framework (exports, digital, environment, energy, and equitable empowerment).^4^ The initiative emphasizes the need to continuously foster new growth drivers, assist Pakistan in better integrating into international industrial cooperation, form competitive industrial clusters, enhance export competitiveness, and achieve self-reliant, balanced, and sustainable development. We must adhere to a people-centered development philosophy, strengthen cooperation in social and livelihood sectors, focus on poverty alleviation, job creation and improving people’s well-being. We should continue implementing the Belt and Road Innovation Action Plan, meticulously construct the China-Pakistan Digital Corridor, and ensure that green development remains the foundation of CPEC construction. As a vital component of the Belt and Road Initiative, the corridor’s development should align with the trend of economic globalization, promoting more open and inclusive cooperation to provide robust momentum and broad space for economic growth. This phase reflects the strategic evolution of CPEC from “construction-oriented cooperation” to a “development-oriented platform.”

In April 2015, President Xi Jinping emphasized that CPEC should play a leading role in practical cooperation between the two countries. Centered on the corridor’s development, with Gwadar Port, energy, infrastructure construction, and industrial cooperation as key priorities, a “1+4” cooperation framework was established. The Joint Cooperation Committee (JCC) formed between the Chinese and Pakistani governments coordinates and oversees the implementation of the CPEC. The JCC will fully leverage its role by regularly convening meetings and working group sessions under its framework to deepen coordination and cooperation. Regular exchanges of experiences in economic development will be conducted to support the high-quality development of the CPEC. The Committee will hold periodic meetings to assess progress, adjust priorities, and provide policy guidance for the corridor strategy. the JCC and reaffirmed the government of Pakistan’s resolve to offer uniform regulations and a favorable atmosphere for Chinese nationals and businesses operating in Pakistan. The JCC also emphasized the importance of the major energy and infrastructure development projects, which are now underway and provide Pakistan numerous chances for socioeconomic growth. The relevant ministries and departments of both countries have established a cooperation mechanism to coordinate the development of CPEC and jointly formulated the Long-Term Plan for CPEC (2017-2030) in accordance with the agreement reached between Premier Li Keqiang and then-Pakistan Prime Minister Nawaz Sharif.

The plan states that short-term projects must be completed by 2020. Major obstacles to Pakistan’s economic and social development will essentially be removed by 2020, when the CPEC aims to take on its original form and begin to accelerate economic progress for both nations. The mid-term project is to be completed by 2025, By 2025, the construction of the CPEC is expected to be nearly finished, the industrial system will be nearly finished, key economic functions will be implemented holistically, the standard of living for those living along the CPEC will have significantly improved, regional economic development will be more balanced, and all of Vision 2025’s objectives will have been met. Long-term projects must be completed by 2030. By 2030, the CPEC project is expected to be completed, the endogenous mechanism for sustainable economic growth will be established, the CPEC’s contribution to promoting economic growth in South Asia and Central Asia will be fully realized, and South Asia will develop into an international economic zone with a global impact. The plan will provide the macro guidance for implementation of CPEC in the next phase, and the plan would be adjusted based on the real situation as well as the consensus between the two parties during its implementation in the future.^5^ The China-Pakistan Economic Corridor has evolved into a relatively mature cooperation mechanism under the Belt and Road framework, encompassing a comprehensive investment plan covering energy projects, road and rail connectivity, infrastructure upgrades, and industrial park development, with substantial progress achieved. By the end of 2020, the transportation and energy corridors under the CPEC comprised 70 projects, with 46 either under construction or completed. The early harvest objectives have been largely achieved, with some delivered ahead of schedule.^6^

#### Medical Assistance Services:

Long Term Plan for CPEC (2017-2030) Provide medical assistance services in more places within the CPEC coverage and upgrade existing medical facilities based on actual needs.^7^ The Action Plan for Building a Closer China-Pakistan Community with a Shared Future for a New Era (2025–2029) between the Government of the People’s Republic of China and the Government of the Islamic Republic of Pakistan proposes to continue cooperation in areas such as healthcare, agriculture, education, climate change response, and disaster prevention and mitigation. More “small yet beautiful” livelihood projects will be developed across Pakistan to enhance the sense of fulfillment among the people of both countries regarding China-Pakistan cooperation.^8^ This underscores that healthcare cooperation constitutes a vital component of people-centered cooperation, integrated into the ongoing agenda for advancing the social dimension of the China-Pakistan Economic Corridor (CPEC). While the Action Plan, as a high-level strategic roadmap, does not feature a dedicated healthcare chapter, it positions healthcare collaboration within the broader context of the People’s Livelihood Corridor and the Technology Corridor. Healthcare cooperation encompasses primary healthcare services, scientific research collaboration, and talent development. As an integral part of CPEC, it will contribute to the objectives of constructing a high-quality corridor and enhancing people’s livelihoods.

Pakistan lacks a comprehensive healthcare system and medical insurance framework. Hospitals are categorized as either public or private. Public hospitals charge low fees and primarily serve ordinary citizens. Private hospitals offer relatively better equipment and medical standards but impose high costs. Major Pakistani cities have both national hospitals and private clinics, ensuring treatment for common illnesses. However, healthcare conditions in rural and remote mountainous areas remain relatively poor. In some remote areas of Pakistan, hospitals even lack stable electricity supply, severely hindering critical medical procedures like surgeries. Additionally, outdated medical communication infrastructure leads to delayed information transfer, making it difficult to implement modern healthcare services such as telemedicine consultations.

This further widens the gap in medical standards between Pakistan and developed nations as well as neighboring developing countries.^9^ Pakistan’s nationwide water supply system is extremely outdated and poorly maintained, with severe water contamination and bacterial levels significantly exceeding safety standards. Compounded by widespread public ignorance of basic hygiene practices, the country frequently experiences outbreaks of infectious diseases such as hepatitis. During summer months, consuming contaminated food or water readily triggers gastrointestinal illnesses, with major infectious diseases including cholera, dengue fever, and malaria.^10^ Pakistan’s healthcare industry is underdeveloped, with overall medical standards remaining relatively low. The number of hospitals is severely inadequate for its vast population. The country lacks a comprehensive healthcare system and medical insurance framework. Private hospitals charge exorbitant fees but offer comparatively better medical equipment, services, and standards. Public hospitals and private clinics, however, operate under poor sanitary conditions, capable only of treating common ailments, and suffer from a severe shortage of medical equipment.^11^ Pakistan’s medical equipment industry is facing severe challenges. Take Sialkot as an example: the region suffers from severe power shortages, with daily outages exceeding 20 hours. Within two weeks, over 500 medical device factories halted production, resulting in 200,000 job losses. Due to production stoppages, Pakistan could not deliver medical devices on time, creating a supply shortage domestically. Many local buyers began sourcing medical equipment from India, China, and Taiwan.

Pakistan’s healthcare system suffers from severe inadequacies and insufficient medical benefits, with only 0.7% of its gross national product allocated to national healthcare welfare. In principle, the Pakistani government provides free healthcare, but in practice, the system has gradually been privatized over the past two decades. In other words, most ordinary citizens have lost their basic medical insurance. The pharmaceutical market is monopolized by large pharmaceutical companies, leading to skyrocketing drug prices that ordinary people cannot afford. Public healthcare facilities face shortages of essential medications and high prices, leaving the poor unable to afford them. Furthermore, exorbitant tuition fees prevent children from impoverished families from attending medical school, with women facing additional discrimination. Women constitute over half of Pakistan’s population, yet societal traditions remain deeply conservative. The scarcity of female doctors and severe shortage of specialists in fields like gynecology further exacerbate national health challenges.^12^

Pakistan has some health insurance plans, but coverage is limited. Most people still rely on out-of-pocket payments or social security programs to cover medical expenses.^13^ Particularly vulnerable groups such as farmers and low-income workers remain excluded from effective medical insurance systems. Even where some insurance programs exist, issues like low reimbursement rates and cumbersome procedures persist, forcing patients to shoulder substantial medical expenses when they fall ill. Consequently, the phenomena of falling into poverty due to illness and relapsing into poverty after recovery remain prominent.^14^ Pakistan has a certain number of healthcare professionals, but shortages of doctors and nurses may exist in some regions.^15^

#### Medical Education:

The training cycle for medical professionals is lengthy and costly, yet Pakistan has relatively insufficient investment in medical education. The number of domestic medical schools is limited, and teaching resources are unevenly distributed, primarily concentrated in urban areas. This prevents many promising students from accessing high-quality medical education, and even fewer graduates are willing to return to serve in grassroots communities after graduation. Furthermore, Pakistan faces a significant brain drain of medical professionals, including doctors and nurses, due to poor working conditions and low compensation, exacerbating the domestic shortage of healthcare personnel. Pakistan’s medical resources are disproportionately concentrated in urban areas, particularly large cities. Urban medical institutions enjoy relatively abundant equipment and personnel, while rural and remote areas suffer from rudimentary facilities and a scarcity of specialized healthcare workers. This imbalance forces rural residents to travel long distances to urban centers for medical care, increasing patients’ financial burdens and potentially worsening conditions due to delayed treatment. For instance, common illnesses often go untreated in rural areas, allowing them to progress into more severe conditions.^16^

### Pakistan Academy of Surgical & Medical Specialists

As society continues to develop and progress, people are no longer satisfied with merely surviving and filling their stomachs. Instead, they place greater emphasis on enhancing their quality of life. Healthcare has become a vital safeguard for human well-being.^17^ On May 31, 2018, Lanmer International Medical Group and representatives of Pakistani medical experts jointly established the Greater China Branch of the Pakistan Academy of Surgical and Medical Specialists. Actively responding to the Belt and Road development strategy, the group has engaged in medical exchange and cooperation with Pakistan. This initiative not only provides opportunities for doctors from both countries to exchange knowledge and learn from each other, but also offers a broad platform for the internationalization of domestic medical technology.^18^ In December 2019, the Gwadar Port Hospital in Pakistan—a medical project symbolizing China-Pakistan friendship—officially commenced construction with Chinese assistance. The hospital’s development has significantly enhanced local healthcare standards, created employment opportunities, and substantially improved living conditions, making it a project that profoundly impacts the lives of local residents.^19^

In 2020, the COVID-19 pandemic swept across the globe, instantly transforming the way people around the world lived. During the pandemic, China repeatedly provided Pakistan with urgently needed medical supplies, including testing kits, protective gear, and ventilators, and dispatched medical expert teams to assist in the fight against the virus. This support safeguarded the health of the Pakistani people and saved countless lives.^20^ China-Pakistan cooperation in healthcare extends far beyond this. In December 2020, Neusoft Medical and Pakistan Power Engineering Automation Company formally signed a cooperation agreement to engage in extensive collaboration across high-end medical imaging technology, artificial intelligence, and training education. This initiative represents another milestone in bilateral healthcare cooperation, significantly enhancing Pakistan’s medical imaging diagnostics capabilities and talent development standards while bolstering Liaoning Province’s efforts to build the “Health Silk Road.”^21^

#### Covid19 Vaccine supply by China:

On February 1, 2021, the first batch of COVID-19 vaccines provided by the Chinese government as foreign aid arrived in Pakistan.^22^ The Chinese Consulate General in Lahore funded the upgrade of the Neonatology Unit and Central Oxygen System at Lady Aitchison Hospital through a Chinese special grant.^23^ At the 2022 International Medical Conference in Islamabad, Chinese medical AI enterprises signed cooperation agreements with Pakistani institutions to jointly advance the application of AI retinal imaging technology in early screening for chronic diseases in Pakistan, thereby enhancing the efficiency of primary-level chronic disease diagnosis. This initiative involves the local promotion and technology transfer of portable retinal cameras and AI-powered diagnostic solutions.^24^ Pakistan also received over 100,000 doses of hepatitis A vaccine produced by China’s Sinovac Biotech for adults and children during a ceremony held at Pakistan’s Ministry of National Health Services.^25^ In early 2022, significant breakthroughs were achieved in traditional Chinese medicine research, with China Tang’s International Education Group signing a memorandum of understanding with a Pakistani institution.^26^ In 2022, the China-Pakistan Medical Association and the China Academy of Chinese Medical Sciences jointly established the Pakistan-China Traditional Medicine Alliance in Jinan, China.

### China Pakistan Friendship Hospital at Gawadar

This alliance will promote research, professional training, and the application of modern technologies in the field of traditional herbal medicine to strengthen cooperation between the two parties.^27^ The China-Pakistan Friendship Hospital is located in Gwadar, Balochistan Province, Pakistan. Construction of the hospital in Gwadar, Balochistan Province, Pakistan was completed in December 2023. It officially commenced operations in May 2024 and has since provided medical services to 150,000 local patients.^28^ The establishment and operation of the China-Pakistan Friendship Hospital have significantly alleviated the challenges faced by the Gwadar region, including scarce medical resources, rudimentary healthcare facilities, and limited diagnostic and treatment options. From January 22 to 23, 2025, the emergency project for congenital heart disease treatment in Pakistan was successfully completed,^29^ This project’s technical cooperation in congenital heart disease treatment exemplifies another “small yet impactful” people-oriented initiative under the China-Pakistan Economic Corridor.

In April 2025, Pakistan and China agreed to strengthen bilateral cooperation in the healthcare sector, focusing on pharmaceutical manufacturing, digital healthcare, and medical technology,^30^ This marks a new phase in bilateral health cooperation. Pakistan and China will strengthen collaboration on cancer at the China-Pakistan International Cancer Research Conference 2025 (CCHIO 2025).^31^ Through this collaboration, Pakistan has taken a significant step forward in advancing cancer research and treatment. Two hundred Chinese companies participated in the fourth Health, Engineering, and Minerals Show (HEMS) held in Lahore from April 17 to 19, 2025.^32^ This exhibition primarily focused on agriculture, industry, healthcare, and investment, aiming to further strengthen economic cooperation between Brazil and China in key sectors.

## METHODOLOGY

In the context of the CPEC, this study looks at the transfer of medical technology and the development of Pakistan’s health system’s capabilities. It identifies the difficulties faced in health system transformation while also exposing effective methods through the combination of qualitative document analysis and comparative policy evaluation.

The data and materials in this review are sourced from multiple reliable channels, including the Pharmacopoeia of the People’s Republic of China and official policy documents from Chinese government agencies such as the State Council, the National Development and Reform Commission, the Ministry of Foreign Affairs, and the National Health Commission. Reports and guidance documents from international organizations such as the World Health Organization (WHO), the Government of Pakistan Ministry of Planning, the Economic and Commercial Office of the Embassy of the People’s Republic of China in the Islamic Republic of Pakistan, and the National Agency for International Development Cooperation were incorporated into the research scope. Additional academic research and policy documents were provided by institutions such as the China Association for Science and Technology and Jianpei Technology. Relevant media coverage was sourced from outlets including Pakistan Today, China Daily, Xinhua News Agency, and China Economic Net. When selecting materials, we comprehensively evaluated their credibility, publication date, and applicability to medical technology transfer and health system capacity building.

### Medical Technology Transfer:

Compared to Pakistan’s population size, the country faces severe challenges due to insufficient medical personnel and facilities. Over 90% of its medical devices and diagnostic equipment are imported from the United States, the United Kingdom, Germany, and China, costing approximately $1.1 billion annually to meet domestic healthcare needs. Against this backdrop, some Chinese companies are focusing on technology transfer and deepening localized production.^33^ Hangzhou Aiche Medical Technology Co., Ltd. (a subsidiary of Boyue Group) and Pakistan’s Laboratory Diagnostic Systems (LDS) will engage in in-depth collaboration in the Point-of-Care Testing (POCT) sector. This marks a significant step toward localizing Pakistan’s medical device industry, injecting new momentum into elevating local healthcare standards and fostering economic growth. Tianjin First Central Hospital, the Management Office of Tianjin Medical Association, and the Lahore Medical Association of Pakistan have also signed agreements to deepen cooperation in public health, clinical medicine, and traditional medicine.

The three parties will engage in information sharing and personnel exchanges, including mutual dispatch of medical professionals and researchers for short-term training or long-term academic visits. They will jointly organize specialized technical training and healthcare management programs, conduct collaborative research aligned with mutual interests, publish research findings together, and jointly advance the commercialization of research outcomes.^34^ The trilateral collaboration demonstrates that physician exchange programs are significantly expanding through the Belt and Road International Medical Education Alliance and the Belt and Road Medical Device Innovation and Application Alliance.

Pakistan holds a distinct advantage in medical consumables such as surgical instruments, with demand for high-value medical products steadily increasing. The Pakistani government places great emphasis on the international development of the surgical instrument industry. China possesses a comprehensive medical product supply chain and holds comparative advantages in high-end medical devices such as imaging equipment and diagnostic products, while demonstrating strong willingness to engage in relevant cooperation with Pakistan. The Pakistani government has introduced a series of incentive measures, including tax exemptions, tariff reductions, land use support, and streamlined approval procedures, aimed at providing comprehensive support and assistance for relevant cooperation between China and Pakistan.^35^ On January 17, 2025, memorandums of understanding (MoUs) worth approximately $1.6 billion were signed at the China-Pakistan B2B Matchmaking Event for Medical Equipment and Surgical Instruments.^36^ Aimed at guiding more Chinese enterprises to participate in trade and establish joint ventures in Pakistan’s medical device sector.

On January 21, 2021, the China-Pakistan Traditional Chinese Medicine Cooperation Center was launched. The ceremony was conducted simultaneously online and offline, with venues set up at the First Affiliated Hospital of Hunan Medical University in China and in Karachi, Pakistan. The official launch of the China-Pakistan Traditional Chinese Medicine International Cooperation Center will undoubtedly exert a positive and far-reaching impact on promoting the high-quality development of traditional Chinese medicine industries in both countries. Leveraging this international platform, both sides will strengthen communication, personnel exchanges, and scientific cooperation. They will deepen research and application in relevant pathologies and therapeutic techniques, thereby enhancing mutual learning and complementing strengths in traditional medicine between China and Pakistan. The establishment of this center will further deepen bilateral cooperation in traditional Chinese medicine and related fields, enabling more achievements to benefit the peoples of both nations.^37^

In November 2024, a multi-hospital teleconference connecting Gilgit-Baltistan, Pakistan, and Kashgar, Xinjiang, China, was successfully conducted at the National Regional Medical Center of Kashgar First People’s Hospital (Shufu Hospital, Affiliated Kashgar Hospital of Sun Yat-sen University). During the meeting, both parties engaged in in-depth discussions on leveraging the Shanghai Cooperation Organization Hospital Cooperation Alliance to enhance medical services, teaching, research, and the comprehensive advancement of translational applications in areas such as liver echinococcosis diagnosis and treatment technologies, as well as medical imaging technologies. Furthermore, guided by a spirit of friendly cooperation, both sides jointly signed a Memorandum of Understanding to jointly promote the in-depth development of international exchange and cooperation in the medical field.^38^ On April 30, 2025, Pakistan and China agreed to strengthen bilateral cooperation in the health sector, with particular focus on pharmaceutical manufacturing, digital healthcare, and medical technology.^39^ The collaboration provides ample resources for numerous Pakistani pharmaceutical companies reliant on Chinese raw materials, while also advocating for joint ventures and public-private partnerships to facilitate the local production of active pharmaceutical ingredients in Pakistan.

### Production of Pharmaceuticals Raw Material

Pakistan possesses significant opportunities in promoting telemedicine. The country is seeking cooperation with China’s private sector to introduce pharmaceutical technologies from China, emphasizing the production of raw materials for essential medicines within Pakistan.^40^ Pakistan has invited Chinese medical companies to establish production bases in Pakistan, aiming to promote investment and medical innovation.^41^ Pakistan’s telemedicine initiative has achieved a significant breakthrough, particularly in bridging primary and secondary healthcare systems. Through online medical services, especially in remote areas, people can now access timely disease prevention.

On May 23, 2025, the “China-Pakistan Traditional Medicine (Herbal Medicine) Online Exchange Meeting” was successfully held. This online exchange meeting opened up new avenues for deepening traditional medicine cooperation between China and Pakistan.^42^ The Pakistani government encourages Chinese enterprises to invest in the country and is promoting specialized legislation on traditional Chinese medicine, aiming to provide a favorable investment environment for Chinese companies.

#### Capacity Building:

In May 2023, Gansu University of Chinese Medicine, the Pakistan Institute of Health Services, and the China Acupuncture Center and Clinical Laboratory in Islamabad reached an agreement to collaborate on cultivating TCM professionals in Pakistan and promoting TCM clinical diagnosis and treatment. Beyond acupuncture and moxibustion, strengthening regulatory frameworks in Pakistan is imperative for the project’s smooth implementation. Consequently, the university has initiated collaboration with Pakistani regulatory bodies to advance the nation’s legislative process for traditional Chinese medicine.^43^ The number of traditional Chinese medicine practitioners in Pakistan is limited, necessitating more training programs to cultivate professionals in this field, thereby bringing greater benefits to the Pakistani people.

On July 1, 2024, a delegation from the Pakistani medical team arrived at the Third Hospital of Hebei Medical University in Shijiazhuang City, Hebei Province, China, to commence a six-week training program.^44^ The training model involves Pakistani physicians participating alongside hospital specialists in activities such as ward rounds, surgeries, case discussions, and academic lectures. This program plays a significant role in strengthening medical cooperation and knowledge exchange between the two countries. In 2024, the Committee on Science and Technology (COMSTECH) of the Organization of Islamic Cooperation (OIC) collaborated with Hunan University of Chinese Medicine (HUCM) to offer a training program for technical personnel covering various disciplines in medicine and STEM (Science, Technology, Engineering, and Mathematics). Under this collaborative initiative, titled the “COMSTECH-Hunan University of Chinese Medicine (HUCM) Technical Personnel Training Program,” 13 participants from seven countries—including two from Pakistan—received advanced training in medical sciences and technology. The program included instruction in nuclear magnetic resonance (NMR) and magnetic resonance imaging (MRI) techniques, alongside specialized courses on innovations in ethnic medicine (particularly developments in traditional medical practices of Chinese ethnic minorities, including developments in Tibetan, Uyghur and Mongolian traditional medical practices) and traditional Chinese medicine (TCM).^45^ The implementation of this project aims not only to attract more individuals to participate in the fields of technology, education, and science, but also to engage in the promotion of traditional Chinese medicine. It holds significant implications for the exchange of medical technologies between the two countries.

The Traditional Chinese Medicine Practitioner Training Program in Pakistan commenced on August 11, 2025. Program participants will bring traditional Chinese medical techniques back to Pakistan, introduce them to their colleagues, and apply them in disease treatment.^46^ In recent years, as the China-Pakistan Economic Corridor has progressed from its “Phase 1.0” to a high-quality “Phase 2.0 upgrade,” its focus has shifted from early-stage infrastructure and energy projects to industrial cooperation, technology transfer, livelihood improvements, and the full operation of Gwadar Port. Bilateral cooperation between China and Pakistan in the field of traditional medicine has significantly expanded.

### Chinese Investment in Health Sector

In 2025, Rizwan, CEO of Citi Group Pakistan, visited Wuzhen Smart Hospital for an inspection tour. Through on-site visits, the Pakistani delegation gained an in-depth understanding of the advanced technologies and management concepts of China’s first artificial intelligence hospital. They expressed hope to introduce the Wuzhen Smart Hospital model to Pakistan, empowering the rapid development of Pakistan’s healthcare industry. Both parties expressed their commitment to further strengthening international cooperation and jointly exploring the global application and development of the Wuzhen Smart Hospital model. This visit and exchange not only established a high-quality communication channel for Sino-foreign medical technology cooperation but also laid a solid foundation for deeper international collaboration in the future.^47^ In May 2025, Pakistan and China agreed to deepen cooperation in the health sector and established a dedicated Pakistan National Institute of Health (NIH) service desk for Chinese investors. The NIH facilitates Chinese investment in Pakistan’s healthcare sector, focusing on local pharmaceutical production, Traditional Chinese Medicine (TCM), and critical vaccine supply. This collaboration offers potential support for China in technology transfer, capacity building, and long-term supply contracts. This collaboration comes as Pakistan seeks to diversify its healthcare inputs, reduce reliance on Western pharmaceutical imports, and bolster domestic production amid recurring supply disruptions and rising healthcare costs.

### Provision of sustainable patient centered Healthcare services

During his visit to Beijing Anzhen Hospital in 2025, the Prime Minister directed that Pakistan’s upcoming hospitals should prioritize constructing high-quality facilities, emulating the Chinese model. He commended China’s efficient hospital management and emphasized Pakistan’s need to replicate this system to enhance healthcare services. He stressed that Pakistan’s new hospitals must prioritize high-quality medical facilities to ensure the provision of sustainable, patient-centered healthcare services.^48^ The Pakistani government is willing to draw on China’s healthcare system to establish quality and management standards. This demonstrates China’s direct influence on Pakistan’s healthcare governance and institutional development, thereby illustrating China’s indirect role in promoting the rule of law through hospital construction in Pakistan.

The China-Pakistan Friendship Hospital, funded by China, stands as a vital people-oriented assistance project under the China-Pakistan Economic Corridor framework and represents a significant cooperative achievement in the high-quality joint construction of the Belt and Road Initiative. As a newly established comprehensive hospital in the region, the Friendship Hospital features well-defined functional zones and a complete array of departments. It is equipped with advanced facilities including CT scanners, X-ray machines, and medical laboratories. Additionally, it operates a “one-stop service platform” providing free medication and meals to inpatients. Since its operation, the hospital has treated approximately 900 patients daily. with over 60,000 patients treated in family medicine and general outpatient clinics, more than 30,000 pediatric cases managed, over 18,000 emergency cases handled, nearly 16,000 consultations conducted in obstetrics, gynecology, and child health, at least 1,260 babies delivered, over 5,000 patients seen in general and orthopedic surgery, and nearly 600 surgeries performed across all departments.^49^ As a new standard medical institution, the hospital’s operations must comply with Brazilian healthcare regulatory requirements. In practice, it may also encourage local health authorities to enhance their enforcement and oversight capabilities.

### Production of APIs, Vaccines and Medical Devices

China’s management, service procedures, and quality control requirements for foreign-funded hospitals will exert practical pressure on the implementation of relevant Pakistani regulations, thereby promoting greater standardization within the local healthcare sector. Pakistan and China agree to deepen health sector ties and establish a dedicated NIH desk for Chinese investors. Pakistan pledges to fully facilitate Chinese investment in its healthcare sector, with a focus on local pharmaceutical production, Traditional Chinese Medicine (TCM), and critical vaccine supply. Pakistan is committed to creating a favorable environment for Chinese enterprises interested in investing in Pakistan’s healthcare-related industries, including the production of Active Pharmaceutical Ingredients (API), vaccines, diagnostic equipment, and medical devices. The Minister announced the establishment of a dedicated China Affairs Office at Pakistan’s National Institute of Health (NIH). This office will provide one-stop services for all Chinese healthcare enterprises operating in Pakistan or preparing to enter the Pakistani market.^50^

As Pakistan’s key health regulatory body, the NIH has established a dedicated task force for Chinese enterprises, which is expected to enhance institutional transparency and procedural standardization for Chinese companies entering Pakistan’s healthcare market. This initiative will facilitate the development of relevant approval processes, standards, and regulatory mechanisms by Pakistani authorities.

This mechanism reflects institutional-level communication and cooperation between the two parties, serving as a catalyst in the process of advancing the rule of law in governance. Since 2024, multiple Chinese and Pakistani institutions have signed Memoranda of Understanding (MOUs). While not legally binding documents, these MOUs symbolize the initial alignment of institutional frameworks and healthcare practices. Cooperation in areas such as telemedicine and big data applications necessitates regulatory standards for information management, data privacy, and cross-border medical data flows—a requirement that can compel Pakistan to refine its relevant systems. Medical cooperation between China and Pakistan—whether through formal agreements, MOUs, or institutional frameworks formed by technical exchanges—constitutes “soft law” level institutional interaction.

In 2020, China dispatched a medical expert team to Pakistan for healthcare exchanges, sharing epidemic prevention expertise and assisting in refining Pakistan’s pandemic control guidelines. This collaboration undoubtedly influenced the formulation of Pakistan’s prevention protocols and operational documents, providing legal and policy foundations for its pandemic response.

## CONCLUSIONS

Medical technology transfer under the China-Pakistan Economic Corridor framework extends beyond resource allocation and technical cooperation to significantly impact capacity building, healthcare professional training, and regulatory governance in hospitals. This is achieved through multiple channels: strengthening hospital construction projects, introducing diagnostic equipment, providing remote medical guidance, enhancing medical personnel training programs, boosting primary healthcare capabilities, and leveraging China’s indirect regulatory influence on Pakistani hospitals. Health cooperation between China and Pakistan has gradually shifted from “infrastructure construction” to “institutional capacity building,” driving Pakistan’s healthcare system transition from relying on foreign investment to strengthening its own medical emergency response capabilities.

Research indicates that the effectiveness of medical technology transfer does not solely depend on infrastructure construction and equipment introduction. It also requires establishing a healthcare regulatory model compatible with the host country’s institutions. Regulatory systems must be refined, institutions coordinated, and crucially, aligned with the domestic policy environment. The Pakistani government’s adoption of China’s modern hospital management and quality improvement models as benchmarks facilitates the development of Pakistan’s own medical technologies. However, the long-term sustainability of Pakistan’s healthcare model remains contingent upon further refinement of its domestic institutional absorption capacity and legal framework. Moving forward, China-Pakistan healthcare cooperation should prioritize shifting from “hardware export” to “joint institutional development.” This entails strengthening regulatory collaboration mechanisms, enhancing legal safeguards, and promoting coordinated development of medical standards, information systems, and professional certification frameworks.

Overall, the China-Pakistan Economic Corridor requires deepening medical technology cooperation and institutional capacity building. This approach holds promise to become a replicable South-South cooperation model for developing nations.
